# Possibilities of Using Tensiomyography to Assess Early Changes in Muscle Function in Patients with Multiple Sclerosis—Pilot Study

**DOI:** 10.3390/jcm14124212

**Published:** 2025-06-13

**Authors:** Piotr Kurzeja, Katarzyna Ogrodzka-Ciechanowicz, Tomasz Szurmik, Edyta Daszkiewicz, Štefan Madarász, Zuzana Hudakova, Karina Rożek, Karol Bibrowicz

**Affiliations:** 1 Institute of Health Sciences, University of Applied Sciences in Nowy Targ, 34-400 Nowy Targ, Poland; 2 Faculty of Motor Rehabilitation, Institute of Clinical Rehabilitation, University of Physical Culture in Krakow, 31-571 Krakow, Poland; katarzyna.ogrodzka@awf.krakow.pl; 3 Faculty of Arts and Educational Science, University of Silesia, 43-400 Cieszyn, Poland; info@orto-med.com.pl; 4 Department of Orthopaedics and Rehabilitation, University Hospital of Orthopaedics Rehabilitation in Zakopane, 34-500 Zakopane, Poland; edyta.daszkiewicz@uj.edu.pl; 5 SNP Central Military Hospital, Faculty Hospital, 034 01 Ružomberok, Slovakia; stefan.madarasz@ku.sk (Š.M.); zuzana.hudakova@ku.sk (Z.H.); 6 Faculty of Health, Catholic University, 034 01 Ružomberok, Slovakia; 7 Department of Public Policy and Health Sciences, University of the National Education Commission, 30-084 Krakow, Poland; karina.rozek@uken.krakow.pl; 8 Science and Research Center of Body Posture, Kazimiera Milanowska College of Education and Therapy, 61-473 Poznan, Poland; bibrowicz@wp.pl

**Keywords:** tensiomyography, multiple sclerosis, muscle stiffness

## Abstract

**Background**: This study aimed to evaluate the functional status of the biceps and triceps brachii muscles using tensiomyography in individuals diagnosed with multiple sclerosis. **Methods**: The study involved 19 patients with multiple sclerosis (MS) aged 19–46 years and 24 healthy individuals aged 20–25 years. Each person underwent a tensiomyographic examination of the biceps and triceps brachii muscles in both upper limbs. The following indices were analysed: contraction time (Tc), delay time (Td), muscle displacement (Dm), and bilateral and antagonist functional asymmetry index. **Results**: For the biceps brachii muscle, a statistically significant difference in muscle stiffness was observed between the MS and healthy groups. These differences were noted on both the left and right sides (*p* < 0.0001). **Conclusions**: The results of the tensiomyographic examination of the arm muscles in the group of patients with MS showed increased stiffness of the biceps brachii muscle. The functional asymmetry index in the triceps brachii muscle showed a significant difference between the sides, but no differences were noted in the functional asymmetry index between the antagonist muscles.

## 1. Introduction

Multiple sclerosis (MS) is an autoimmune multifactorial disease characterised by the development of demyelinating lesions in various areas of the central nervous system (CNS) [1]. The aetiology of the disease is hypothesised to be the result of a combination of genetic and environmental factors. To date, research has identified over 200 genetic variants that are associated with an increased risk of developing MS [2]. The pathophysiology of MS is believed to involve abnormal adaptive immune responses that traverse the blood–brain barrier, resulting in the attack on the myelin sheaths surrounding neurons [3]. The prevalence of MS has been steadily increasing in recent decades, and current estimates suggest that there are approximately 2.8 million people with the disease worldwide [4]. The course of the disease is characterised by an initial projection–remission phase (RRMS), defined by neurological symptoms and subsequent disability [5]. As the disease progresses, most patients typically experience a progressive decline in physical ability, which is not typically associated with relapses [5,6].

In patients diagnosed with MS, changes in muscle tension and strength may be a characteristic feature of the disease [7]. As demonstrated by Alentorn-Geli et al., changes to muscle mass and tone in MS are frequently associated with a loss of muscle tissue, which can result in an imbalance in muscular strength and endurance [8]. A typical and specific finding in these patients may be a disturbed, asymmetrical gait characterised by uneven steps, which may be due to muscle fatigue and a lack of motor control [9]. Other motor impairments (related to muscle spasticity) and autonomic muscle damage (urinary dysfunction, sexual dysfunction and bowel damage) are among the typical symptoms of the disease as well [10]. Upper limb dysfunction is also common in MS patients. It has been reported that 75 per cent of people with MS encounter difficulties when performing bimanual tasks. This has a detrimental effect on their independence and ability to perform daily activities, thereby reducing their quality of life. Such dysfunction is characterised by a combination of sensory, motor and central disturbances. MS disorders manifest in either unilateral or bilateral forms, with the potential to occur proximally or distally [11].

The diagnosis of MS is based on a thorough medical history, combined with clinical and laboratory examination [5]. It is important to note that disease activity in the first years after MS diagnosis has a significant impact on long-term prognosis [12,13]; thus, it is vital to recognise pathological changes at an early stage of the disease, which enables a tailored and personalised therapeutic approach to be developed [14].

To date, no studies have been conducted that address structural changes in the upper and lower limb muscles of MS patients. Most of the extant literature focuses on the assessment of neuromuscular conduction and its relation to the demyelination process. For this purpose, magnetic resonance imaging (MRI), computed tomography (CT) and electromyography (EMG) are primarily used. However, it appears that tensiomyography (TMG) can also be vital in assessing the electrical activity of fatigued muscles specific to MS patients, revealing how certain accessory motor units are activated [15].

Tensiomyography is a method of assessing the functional state of muscles. It enables the assessment of the contractile properties of muscle fibres after electrical stimulation. The method is based on the diagnosis of functional symmetry, temporal or morphological. It also enables the assessment of muscle synchronisation and the rapid detection of preclinical changes in muscle function [16,17]. To date, tensiomyography has mainly been implemented in sports medicine [18,19], the evaluation of muscular stiffness, involutional alterations in the muscular system in elderly individuals [20], spinal pain syndromes [21] and the early identification of changes in paraspinal muscle function in idiopathic scoliosis. As evidenced by Neamtu et al. and Rusu et al., the phenomenon under discussion also pertains to multiple sclerosis. One notable fact in the context of MS is that one of the consequences of the demyelinating process is altered muscle contraction, which may not be clinically detectable at an early stage [22,23]. Consequently, the use of TMG in the diagnosis of MS may facilitate the early detection of alterations in muscle fibres.

The objective of this study was to evaluate the functional status of the biceps and triceps brachii muscles using tensiomyography in individuals diagnosed with MS.

## 2. Materials and Methods

### 2.1. Study Design

An observational (cross-sectional) study was performed in accordance with the principles of the Declaration of Helsinki and the Strengthening the Reporting of Observational Studies in Epidemiology (STROBE) Statement: guidelines for reporting observational studies [24]. This project was also approved by the Bioethics Committee at the Kazimiera Malinowska College of Education and Therapy in Poznań (No. 003/2019, approval date: 15 February 2019).

### 2.2. Setting

This study was conducted between August and October 2022 at the Neurology Clinic in Ružomberok, Slovakia.

### 2.3. Participants

This study comprised 45 subjects, including 21 patients (10 females, 11 males) with MS and 24 healthy subjects. The subjects with MS were patients receiving MS treatment at the Neurology Clinic in Ružomberok (Slovakia), aged between 19 and 46 years. All patients exhibited independence and ambulatory capacity, with no evidence of disease activity. The comparison group comprised 24 healthy physiotherapy students from the University of Education and Therapy in Poznań (14 women, 10 men).

The following criteria were applied to determine the inclusivity of this study:

1. The diagnosis of MS (MS) was based on a neurological examination, which was conducted by a neurologist, according to the McDonald criteria.
2. The patients were deemed to be clinically stable MS patients, characterised by no worsening or exacerbation of the Expanded Disability Status Score (EDSS) in the six months preceding this study.
3. The patients had no other diseases or injuries to the dominant upper limb.
4. The patients did not participate in any form of rehabilitation in the month prior to the measurements.
5. Written consent to participate in this study was obtained.

This study used a system for examining muscle functional status, which employed the TMB S2 tensiometry method from TMG BMC (Ljubljana, Slovenia). The following indicators were assessed:

1. Tc (ms)—the contraction time, defined as the time interval between the onset of 10% of muscle contraction and the subsequent attainment of 90% of the maximum level;
2. Td (ms)—the delay time, which is measured from the moment of stimulation to the point at which 10% of muscle contraction is achieved;
3. Dm (mm)—muscle displacement, a variable that is associated with Tc and contingent on the elasticity of the muscle tissue. The magnitude of the force exerted by the muscles during explosive movement is found to increase in proportion to the degree to which muscle tone is elevated. Conversely, the force is observed to decrease in proportion to the degree of muscle tone elevation.

The experiment was conducted on the biceps brachii and triceps brachii muscles in the dominant upper extremity, thereby ascertaining the bilateral and antagonist functional asymmetry index.

### 2.4. Outcome Measures

**Figure 1:**
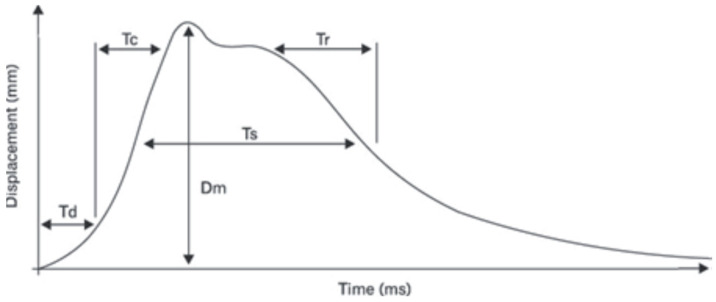
The TMG displacement curve with pertinent indicators. The following terms are used to describe the temporal progression of muscular contraction: delay time (Td), denoting the interval between the electrical impulse and 10% of the contraction; contraction time (Tc), denoting the interval between 10% and 90% of the contraction; sustain time (Ts), denoting the interval between 50% of the contraction and 50% of the relaxation; relaxation time (Tr), denoting the interval between 90% and 50% of the relaxation; and displacement (Dm), denoting the maximum amplitude of the muscle contraction [29].

The TMGTM Science for Body Evolution tensiometry device (TMG-S2 from TMG-BMC d.o.o., Ljubljana, Slovenia) was used in this study. The tensiomyography apparatus includes four constituent components: a pulse-generating unit, electrodes, a mechanical sensor and a control unit with software for data recording and analysis. Surface electrodes must be placed on the belly of the muscle under examination, in line with the direction of the muscle fibres, as close as possible to the centre of its length, at approximately 5 cm. The mechanical sensor is positioned between the electrodes, with its head mounted on a tripod, which facilitates precise manipulation of the sensor’s position and its stabilisation during measurement. The sensor in question can measure the change in the belly’s thickness when stimulated by an electrical impulse. During a TMG test, the magnitude of change in muscle belly displacement in millimetres (mm) and duration in milliseconds (ms) are measured in response to a single electrical stimulus. The measurement process involves the evaluation of muscle response in the context of MS, including Tc, Td, Dm, Tr and Ts. The measurement data is represented by a characteristic time-shift graph. The quantities obtained are shown in Figure 1, in which the displacement (measured in millimetres) is expressed using a percentage scale to illustrate the properties of the individual time variables [25,26,27,28].

### 2.5. Intervention

This study was conducted in a physician’s office between 8:00 a.m. and 2:00 p.m. The subject assumed a seated position in a chair with their arms positioned along the torso. The electrodes were aligned with the course of the bellies of the muscles tested in the dominant upper extremity. The duration of muscle contraction was measured using a single electrical stimulus. Two self-adhesive electrodes were then positioned circumferentially around the TMG sensor. The anode was positioned distally and the cathode proximally, at 20–50 mm from the measuring point. The bipolar electrical stimulation involved a single pulse of direct current with a duration of 1 millisecond.

Two self-adhesive electrodes, each measuring between 2 and 4 cm in diameter, were applied to the subjects’ bodies. The diameter of the electrodes was selected based on the dimensions of the muscles to isolate the contraction of a specific muscle and prevent the activation of adjacent muscles. The electrostimulator delivered a single 1 ms rectangular pulse to the electrodes to induce muscle contraction via the transcutaneous route. The pulse power was then increased in 10 mA increments until the peak response of the muscle contraction was achieved. Typical maximum responses were observed between 40 and 90 mA. Pauses of 10 s were maintained between each stimulation pulse to minimise the effects of fatigue. The sensor’s location was determined in accordance with the TMG scheme; specifically, it was placed on the thickest part of the muscle. Where it was deemed necessary, the sensor application site was subsequently changed to achieve the optimal mechanical response. The sensor was positioned at approximately 5 cm from the centre of the electrodes, with its adhesive surface placed on the skin at this point.

**Figure 2:**
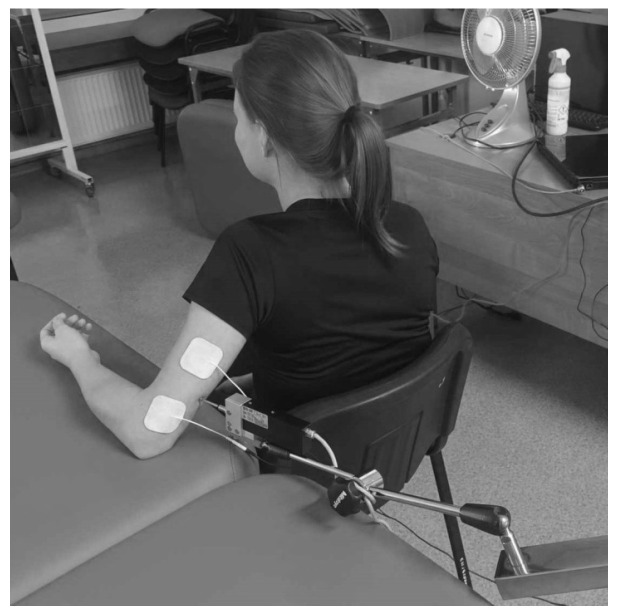
Tensiomyographic examination of the triceps brachii muscle (own source).

**Figure 3:**
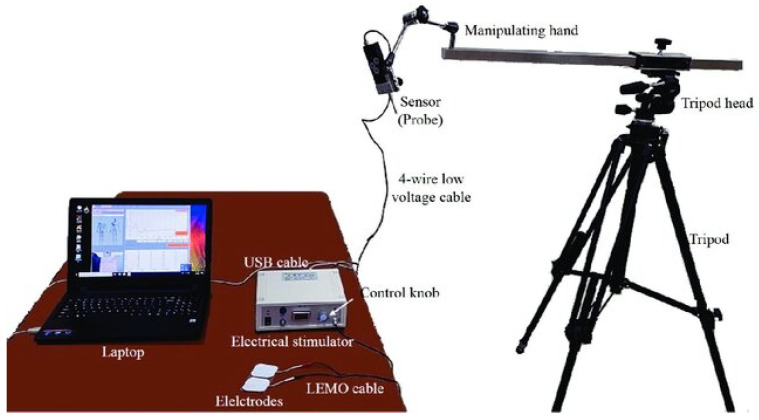
Tensiometry device (own source).

The digital TMG signal was directly acquired from the sensor at a sampling rate of 1 kHz. After measurement, the TMG signal data was stored on a computer disk. The maximum values obtained in the two measurements were stored and averaged for future analyses. The maximum stimulation amplitude was defined as the minimum amplitude required to elicit the greatest muscle displacement (Dm) (Figure 2 and Figure 3).

### 2.6. Statistical Analysis

The statistical analysis of the examination results was performed using MedCalc software (MedCalc^®^ Statistical Software, version 23.1.3 (MedCalc Software Ltd., Ostend, Belgium; https://www.medcalc.org; accessed on 2 January 2025). The Kolmogorov–Smirnov test was used to assess the distribution of the studied variables. A standard descriptive analysis was presented using the mean values (X) and their standard deviations (SDs). The differences were calculated by means of Student’s *t*-test for independent groups.

## 3. Results

The initial plan was to recruit 45 subjects for this study. This included 21 patients with MS (10 females and 11 males), as well as 24 healthy subjects. However, only 43 subjects qualified for this study, as 2 of the MS patients declined participation. Following a comprehensive analysis of the available data, a total of 19 patients (10 females and 9 males) aged between 19 and 46 years (mean age = 31) were included in the MS group. In contrast, 24 participants (14 females and 10 males aged between 20 and 25 years; mean age = 22) were selected for the healthy group. The characteristics of the study subjects are shown in Table 1.

**Figure 4:**
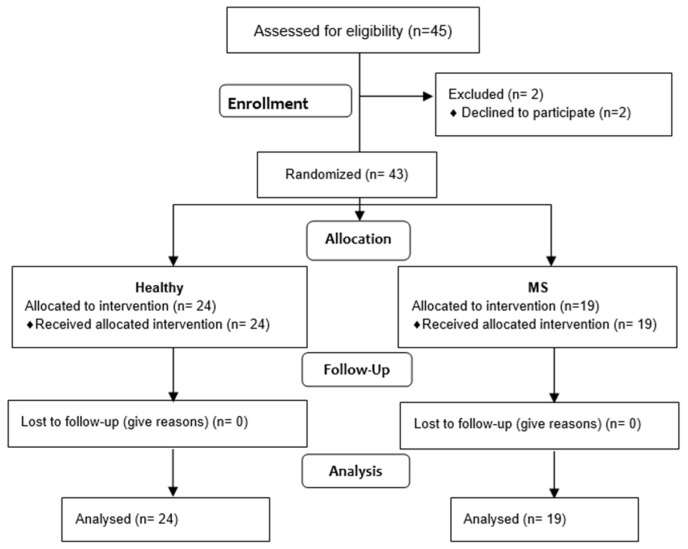
Flow diagram.

The process of qualification for this study is defined as shown in Figure 4.

An analysis of the results of radial displacement (Dm) tests revealed that the variation in muscle stiffness between the two groups depended on the specific muscle studied. For the biceps brachii muscle, a clear, statistically significant difference in muscle stiffness was observed between the MS and healthy groups. These differences were noted on both the left and right sides. In individuals with MS, the magnitude of radial displacement was significantly smaller, indicating higher muscle stiffness. For the triceps brachii muscle, the displacement values were lower compared to the healthy group, though these differences did not reach statistical significance (Table 2).

A study was conducted to analyse the results of Tc muscle reaction time tests. The results demonstrated heterogeneous variation depending on the muscles studied. For the biceps brachii muscles, a slightly faster muscle response was observed in the healthy group. However, these differences were not statistically significant. A similar pattern was observed for the triceps brachii muscle, with those diagnosed with MS exhibiting prolonged response times. However, it should be noted that these differences were only statistically significant for the muscles on the right side (Table 3).

An analysis of the results of latency time (Td) tests revealed variation in the results depending on the muscles studied. For the biceps brachii muscles, a clear, statistically significant difference in the studied variable was observed on both sides. Conversely, the triceps brachii muscles exhibited statistically insignificant differences between the study groups (Table 4).

A thorough analysis of the bilateral asymmetry results indicated a comparable development of function in the arm biceps muscles. As for the triceps brachii muscles, however, significant differences were evident between the left and right sides (Table 5).

Nevertheless, a lack of statistically significant variation was observed in the magnitude of functional asymmetry among the antagonistic muscles examined in the respective groups (Table 6).

## 4. Discussion

This study aimed to evaluate the functional status of the biceps brachii and triceps brachii muscles in patients diagnosed with MS using tensiomyography. The authors hypothesised that tensiomyography, a technique that facilitates the early detection of abnormal muscle contraction, would help assess changes in muscle function in patients with multiple sclerosis. Identifying these changes will hopefully make it possible to plan effective physiotherapy for such patients in advance.

For the biceps brachii muscle, a significant difference in muscle stiffness was observed in the MS group compared to the healthy group. In addition, the triceps brachii muscle exhibited prolonged reaction times. Furthermore, an analysis of the results of the delay time (Td) of the biceps brachii and triceps brachii muscles among the MS group and the healthy group demonstrated a discrepancy. In turn, an analysis of the results of the Td of the biceps brachii and triceps brachii muscles demonstrated variability in their formation, depending on the specific muscle studied. Significant disparities were identified in the biceps brachii muscle on the right side. In the case of the triceps brachii muscles, no such differentiation was observed between the results of the MS group and the healthy group.

It has been hypothesised that examining the passive mechanical properties of muscles can provide an additional diagnostic element, related to the assessment of skeletal muscle adaptive processes (e.g., hypertrophy, muscle strength). Using TMG, particularly the interpretation of two parameters—contraction time (Tc) and radial displacement (Dm)—can prove beneficial in conducting this assessment [30].

Mamoei et al. reported that the progression of MS leads to an increased proportion of fast-twitch fibres, resulting in a more rapid onset of fatigue in patients with MS [31]. Rusu et al. observed that a reduction in contraction time (Tc) may be associated with an increased involvement of type II fast-twitch fibres [32]. In our studies, these differences were only visible in the triceps brachii muscle on the right (dominant) side. The average contraction time for the triceps brachii muscle in MS patients was 22.1 ms, while in the healthy group, it was 33.7 ms; the differences were statistically significant (*p* = 0.01).

According to Haff et al., type II muscle fibres are the primary contributors to the generation of maximum force and rapid movement [33]. As demonstrated by García-Manso, an increase in Tc values can also be observed in situations involving muscle fatigue [34]. Some authors believe that elevated Tc values and diminished Dm may be associated with forceful muscular exertion [35,36,37]. In our studies, a similar tendency was observed for the triceps brachii muscle of the dominant hand. The Dm value was 5.4 mm in patients with MS and 10.8 mm in the healthy group (*p* < 0.0001). Nonetheless, the authors did not confirm these relationships in relation to the Tc value. Thus, in the MS group, the Tc for this muscle was 22.1 ms; in the healthy group, it was 33.7 ms (*p* = 0.01).

There is a dearth of studies on the correlation between muscle weakness in MS and muscle strength values based on the MMT (manual muscle testing) scale. Compston et al. noted that 52% of a group of 301 patients suffered from muscle weakness [38]. Hoang et al. demonstrated muscle weakness in 72% of patients out of a total of 142 patients with MS [39].

As shown in the relevant literature, including the works of Chung et al. and Thoumie et al., muscle weakness in MS is associated with decreased performance, functional status and general fatigue [40,41]. This results in challenges associated with performing daily activities and a diminished quality of life. A reduction in muscle fibre size appears to be a significant component in the development of muscle weakness. Furthermore, patients diagnosed with MS exhibit multiple pathological mechanisms that underlie changes in muscle function, which are not entirely predictable. The unpredictability of the condition is primarily attributable to the co-occurrence of both pre-existing lesions and new lesions. This observation signifies the dynamic evolution of these diseases and the non-linear changes in disease progression. It has been observed that insidious and unnoticeable changes at the onset of the disease are a characteristic feature of MS [42]. As demonstrated by Kent-Braun et al., a decrease in the percentage of type I fibres and an increase in type II fibres was observed in the biopsy of the mTA (medial temporal artery) muscle in patients with MS [43]. Considering the above, the authors performed a comparative analysis of the functional index of bilateral asymmetry and antagonists. The function of the biceps brachii muscles was similar in the MS and healthy groups; however, in relation to the triceps brachii muscle, differences were visible between the left and right sides (*p* = 0.03). Nonetheless, no significant differences were found between the degree of the functional asymmetry for the tested antagonist muscles in both groups. A significant challenge for individuals diagnosed with MS is the prevalence of muscle fatigue and a decline in muscle strength, which can range from 16 to 57% [44]. The predominant aetiological factor appears to be the demyelinating process in the upper central neurons [45]. Consequently, muscle activation in MS is reduced due to a deficiency of motor units [46]. Patients diagnosed with MS appear to exhibit a reduced proportion of type I fibres and an elevated proportion of type II fibres. Changes in the distribution of muscle fibres have been observed to be associated with spasticity and muscle weakness, which occur in most patients diagnosed with MS. Despite the extensive corpus of scientific literature addressing spasticity, further research is required to facilitate a comprehensive evaluation of muscle weakness in MS. One of the principal characteristics that hinders muscle strength in MS patients is muscle weakness.

This, in turn, has a detrimental effect on the day-to-day activities of those affected. Neamtu et al. conducted a study on 13 patients (mean age 38 years) diagnosed with MS who also exhibited clinically detectable gait disturbances. The assessment was conducted using TMG methodology, with the parameters of contraction time (Tc), support time (Ts) and displacement (Dm) being the primary focus of observation. The authors in question observed an escalated level of tension [46].

TMG is a non-invasive way of assessing muscle properties without engaging tendons or requiring joint movement during electrical stimulation. An analysis of two parameters, such as Dm and Tc, can determine muscle behaviour and help provide early information about the possibility of developing musculoskeletal system disorders. This makes it possible for therapeutic intervention to commence early in order to limit the progression of muscle damage and activate neuroplasticity mechanisms. The Dm parameter is also useful for monitoring spasticity and predicting the evolution of muscle tone, which can impair the function of the upper and lower limbs. TMG makes it possible to monitor changes in muscle behaviour over time and can help determine the effectiveness of therapeutic intervention used to maintain the functional state of muscles in patients with MS. Our studies have shown that tensiomyography can be useful in assessing progressive changes in the muscles of MS patients, but further research is required to develop the premises and full indications for the use of this method in tracking muscular system changes that occur in MS. To date, there has been no information in the literature on the use of tensiomyography in the diagnosis and assessment of treatment outcomes in patients with MS. Nonetheless, tensiomyographic assessment may be a valuable addition to the previously used methods for diagnosing and monitoring the development of changes in MS patients.

### Study Limitations

Several limitations should be considered when interpreting the study results. Future studies should qualify study participants with various levels of disability (e.g., mild, moderate, severe) to determine whether this assessment is effective in patients with different degrees of disability. Despite the random order in which the assessment was conducted, we cannot rule out the influence of mental and neuromuscular fatigue, as well as MS-related fatigue, on the results. Assessment of these factors should be included in similar future studies.

## 5. Conclusions

The results of the tensiomyographic examination of the arm muscles in the MS group showed increased stiffness of the biceps brachii muscle.

The functional asymmetry index in the triceps brachii muscle showed a significant difference between the sides, but no differences were noted in the functional asymmetry index between the antagonist muscles.

TMG may be a helpful tool in the early diagnostic assessment of muscle function in patients with MS.

## Figures and Tables

**Table 1:** Anthropometric data.

Variable	SM n = 19	Healthy n = 24
	Mean ± Std.	Mean ± Std.
Weight (kg)	79.4 ± 12.23	81.3 ± 10.11
Height (cm)	174.5 ± 8.45	179.4 ± 8.21
Age (years)	31 ± 1.15	22 ± 2.31
Sex	10 F/9 M	14 F/10 M

n—number of participants; X ± Std.—mean ± standard deviation.

**Table 2:** Radial displacement (Dm, in mm) of biceps brachii and triceps brachii muscles in MS and healthy groups.

Displacement—Dm (mm)						
Group—Side/Muscle	Left Side			Right Side		
	MS X ± SD	Healthy X ± SD	Difference Test *t* *p*-Value	MS X ± SD	Healthy Mean ± SD	Difference Test *t* *p*-Value
Biceps brachii	7.0 ± 4.05	13.4 ± 3.52	t = −5.54 *p* < 0.0001 *	6.1 ± 3.51	10.8 ± 3.18	t = −4.45 *p* < 0.0001 *
Triceps brachii	5.4 ± 3.91	7.1 ± 3.91	t = −1.44 *p* = 1.55	5.4 ± 3.68	6.7 ± 3.88	t = −1.17 *p* = 0.24

MS—multiple sclerosis group, healthy—healthy group; X ± SD—mean ± standard deviation; *—statistically significant.

**Table 3:** Contraction time (Tc, in ms) of biceps brachii and triceps brachii muscles in MS and healthy groups.

Contraction Time—Tc (ms)						
Group—Side/Muscle	Left Side			Right Side		
	MS X ± SD	Healthy X ± SD	Difference Test *t* *p*-Value	MS X ± SD	Healthy Mean ± SD	Difference Test *t* *p*-Value
Biceps brachii	23.4 ± 4.08	24.4 ± 3.58	t = −0.28 *p* = 0.77	22.3 ± 3.08	24.4 ± 6.58	t = −4.45 *p* = 0.20
Triceps brachii	27.6 ± 11.91	22.9 ± 9.29	t = −1.44 *p* = 1.49	22.1 ± 9.19	33.7 ± 20.87	t = 2.45 *p* = 0.01 *

MS—group, healthy—healthy group; X ± SD—mean ± standard deviation; *—statistically significant.

**Table 4:** Delay time (Td, in ms) of biceps brachii and triceps brachii muscles in MS and healthy groups.

Delay Time—Td (ms)						
Group—Side/Muscle	Left Side			Right Side		
	MS X ± SD	Healthy X ± SD	Difference Test *t* *p*-Value	MS X ± SD	Healthy Mean ± SD	Difference Test *t* *p*-Value
Biceps brachii	26.7 ± 3.73	31.2 ± 6.36	t = 2.73 *p* = 0.01 *	26.1 ± 2.86	30.8 ± 7.95	t = 2.43 *p* = 0.01 *
Triceps brachii	23.5 ± 3.19	25.6 ± 8.27	t = −1.06 *p* = 0.29	25.6 ± 6.33	22.8 ± 5.11	t = 1.58 *p* = 0.12

MS—multiple sclerosis group, healthy—healthy group; X ± SD—mean ± standard deviation; *—statistically significant.

**Table 5:** Bilateral asymmetry index (%) of biceps brachii and triceps brachii muscles in test and healthy groups.

Bilateral Asymmetry Index (%)			
Group—Side/Muscle	Left/Right Side		
	MS X ± SD	Healthy X ± SD	Difference Test *t* *p*-Value
Biceps brachii	84.4 ± 8.34	82.0 ± 9.53	t = −0.85 *p* = 0.39
Triceps brachii	69.7 ± 15.41	78.7 ± 11.24	t = −2.17 *p* = 0.03 *

MS—multiple sclerosis group, healthy—healthy group; X ± SD—mean ± standard deviation; *—statistically significant.

**Table 6:** Functional asymmetry index (%) of biceps brachii and triceps brachii muscles in MS and healthy groups.

Functional Asymmetry Index (%)			
Group—Side/Muscle	Biceps/Triceps		
	MS X ± SD	Healthy X ± SD	Difference Test *t* *p*-Value
Left side	74.2 ± 12.22	73.3 ± 11.74	t = 0.21 *p* = 0.83
Right side	68.7 ± 12.89	67.9 ± 12.89	t = 0.15 *p* = 0.88

MS—multiple sclerosis group, healthy—healthy group; X ± SD—mean ± standard deviation.

## Data Availability

All relevant data are contained within our paper.
